# LDH-A promotes malignant behavior via activation of epithelial-to-mesenchymal transition in lung adenocarcinoma

**DOI:** 10.1042/BSR20181476

**Published:** 2019-01-15

**Authors:** Xiao-ming Hou, Shu-qiao Yuan, Da Zhao, Xiao-jun Liu, Xin-an Wu

**Affiliations:** 1 Department of Oncology, The First Hospital of Lanzhou University, Lanzhou 730000, Gansu Province, People’s Republic of China; 2 The First Clinical Medical College of Lanzhou University, Lanzhou 730000, Gansu Province, People’s Republic of China; 3 Department of Medical Laboratory, The First Hospital of Lanzhou University, Lanzhou 730000, Gansu Province, People’s Republic of China; 4 Department of Pharmacy, The First Hospital of Lanzhou University, Lanzhou 730000, Gansu Province, People’s Republic of China

**Keywords:** EMT, LDH-A, LUAD, Prognosis, Tumor progression

## Abstract

Lactate dehydrogenase A (LDH-A) is a key enzyme during glycolysis, which increases the synthesis of related proteins and has elevated activity in cancer cells. The role of LDH-A in lung adenocarcinoma (LUAD) progression was investigated in the present study. Expression levels of LDH-A were assessed in LUAD samples, and the relationship between LDH-A expression status and the prognosis of LUAD patients was confirmed. The effect of LDH-A on proliferation, invasion, migration, and colony formation of cancer cells was assessed. We further determined the role of LDH-A in tumor growth *in vivo* by using xenograft LUAD tumor models. The potential mechanism of LDH-A promotion in LUAD progression was explored. LDH-A showed an abnormally high expression in LUAD, which is closely associated with poor prognosis in patients with LUAD. In *in vitro* experiments, silencing LDH-A expression in LUAD cells could effectively inhibit proliferation, invasion, migration, and colony formation of cancer cells. In *in vivo* experiments, tumor growth was markedly inhibited by LDH-A silencing in a xenograft model of LUAD. Notably, LDH-A could also promote tumor progression by regulating epithelial–mesenchymal transition (EMT)-related molecules. LDH-A can promote the malignant biological behaviors of LUAD cells, and thus can be a potential target for LUAD treatment.

## Introduction

Lung cancer is a malignant tumor with the highest morbidity and mortality worldwide. Among all primary lung cancers, lung adenocarcinoma (LUAD) is the main pathological subtype [1]. In recent years, with the extensive application of targeted medicine in the clinic, the survival time of LUAD patients has been extended. Nonetheless, a majority of LUAD patients are diagnosed at advanced stages; as such, the 5-year survival rate remains <15% [2]. Therefore, searching for molecular and therapeutic targets related to LUAD prognosis in patients is an area of great research interest.

Several studies have indicated that the cancer cell is a specialized cell type, characterized by active proliferation and vigorous growth. These cells possess some features that either cannot be seen, or can rarely be seen in normal cells [3]. Typically, sufficient energy and nutrient supplies are required to guarantee cancer cell growth, proliferation, infiltration, and metastasis. However, cancer cells have poor mitochondrial function and oxidative phosphorylation impairments, which preferentially generate energy through glycolysis. This result in abundant lactic acid production. This phenomenon is referred to as the Warburg effect, which is prevalent in cancer cells [4].

Enhanced glycolysis stimulates increased gene expression of some key enzymes, increased synthesis of related proteins, and elevated activities in cancer cells. Among them, lactate dehydrogenase A (LDH-A) is a key enzyme during glycolysis. Abnormal LDH-A expression has been discovered in multiple solid tumors, which is an indispensable molecule that promotes tumor progression [5–7]. Therefore, the present study aimed to explore LDH-A expression in LUAD, as well as the relationship between LDH-A and the malignant biological behaviors of LUAD.

## Methods

### Tissue specimen and cell culture

Fifty LUAD tissues and matched para-carcinoma tissue pairs were surgically resected and collected from The First Hospital of Lanzhou University from 2008 May to 2018 May. All patients were confirmed pathologically and had complete clinical data. There were 31 males and 19 females, with ages ranging from 40 to 75 years (average, 55.8±10.4 years). The tumor node metastasis (TNM) staging system, jointly formulated by American Joint Committee on Cancer (AJCC)/Union for International Cancer Control (UICC), was used for clinical staging. All patients were naive to chemotherapy, targeted therapy, or biological therapy before surgery. All patients or their family members provided informed consent. The present study was approved by the Ethical Committee of The First Hospital of Lanzhou University.

Human LUAD cell lines A549, H1975, H1299, and SPCA1, as well as the normal human bronchial epithelial cell line (HBE), were purchased from the Shanghai Branch of the Chinese Academy of Sciences. All cells were cultured in the 1640 medium containing 10% fetal bovine serum (FBS), 100 U/ml penicillin, and 100 μg/ml streptomycin (Invitrogen, Carlsbad, CA, USA). Subsequently, cells were cultured in an incubator at 37°C and 5% CO_2_. The medium was replaced every 3 days.

### Immunohistochemistry (IHC) and evaluation

LDH-A expression in cancer tissues and matched para-carcinoma tissues was detected through the IHC method (avidin-peroxidase staining technique). Briefly, tissue paraffin blocks of LUAD patients were collected and sliced into 4 μm-thick sections, followed by xylene deparaffinization and gradient alcohol dehydration (at the concentrations of 95%, 80%, and 75% for 5 min each time). The endogenous peroxidase activity was then blocked using 0.5% H_2_O_2_, followed by rinsing with sterile distilled water for 5 min. Following this, the glass slides were boiled for 10 min in citrate buffer solution (pH=6) to extract the antigen. After washing with phosphate buffer solution (PBS), non-specific binding sites were blocked with normal goat serum for 15 min. Afterwards, the sections were incubated with mouse monoclonal LDH-A antibody (Proteintech, Chicago, IL) at 4°C overnight. Additional glass slides were incubated with PBS overnight as a negative control. Finally, IgG was added as the secondary antibody at 37°C for incubation. The 3,3′-diaminobenzidine (DAB) was used for color developing, and hematoxylin was added as a counterstain.

The LDH-A expression level was calculated using the semi-quantitative rating system. Negative staining was counted as 0 points, weak staining as 1 point, moderate staining as 2 points, and strong staining as 3 points. The stained cancer cell percentage was calculated based on the following method: 0 points (0–5%), 1 (6–25%), 2 (26–50%), 3 (51–75%), and 4 (≥76%). Finally, the product of the cancer cell staining score and the corresponding percentage score were used as the final semi-quantitative score (0–12 points). In the present study, 6–12 points were defined as high LDH-A expression.

### Western blotting

The protein levels were analyzed by Western blotting. Protein was collected and extracted from cells using a protein lysis buffer. Afterwards, equivalent amounts of cell lysate were isolated on 10% sodium dodecyl sulfate-polyacrylamide gel electrophoresis (SDS-PAGE), which was then transferred onto a polyvinylidene fluoride (PVDF) membrane. The membranes were mounted and incubated with primary antibody at 4°C overnight, followed by incubation with goat antirabbit IgG (1:1000; Bioworld Biotechnology Co., Ltd., Nanjing, Jiangsu) as secondary antibody. Finally, the band intensity was analyzed by optical density. In the present study, the primary antibodies used included: anti-LDH-A antibody, anti-E-cadherin, anti-vimentin, anti-N-cadherin, anti-Snail, and anti-ZEB1 antibodies (Abcam, Cambridge, MA, USA).

### Cell transfection

The endogenous LDH-A expression in the LUAD cell line was silenced using the small interference RNA (siRNA) technique. Cells at the logarithmic phase were collected and inoculated into the six-well plate (Corning Costar, Corning, NY, USA) at a density of 1×10^6^ cells/well. The cells were further cultured until 50% confluence, followed by transfection. The Lipofectamine RNAimax reagent (Invitrogen, Carlsbad, CA, USA) was used for cell transfection in strict accordance with the manufacturer’s instructions. The target sequences of LDH-A were shown as follows: siRNA-1, 5′-AAUAACUAGCAGCUUUAUGACC AUAAAGCUGCUAGUUAUUAU-3′ and siRNA-2, 5′-AAUUAAGACGGCUUUC UCCCUGGAGAAAGCCGUCUUAAUUUG-3′. The untreated LUAD cells were used as the normal control group (NC group). The silencing efficiency was verified through Western blots prior to the subsequent experiment.

### Cell proliferation assay

The CCK-8 kit (Yesen, Shanghai, China) was used to determine the cell proliferation capacity. Cells were inoculated into a 96-well plate at a density of 1×10^5^ cells/well for 24 h, 48 h, 72 h, and 96 h, respectively. Subsequently, CCK-8 solution (10 μl/well) was added, and cells were incubated at 37°C for 2 h. Finally, the optical density at 450 nm was measured using a cell reader.

### Transwell assay

Cell invasion capacity was determined through the transwell assay. In brief, 3×10^4^ cells in the 1640 medium containing 2% FBS were inoculated into the upper transwell chamber (BD Biosciences, San Jose, CA, USA). Medium containing 10% FBS was then added into the lower chamber. After 48 h, cells were fixed in 4% paraformaldehyde, stained with 0.1% crystal violet, counted under a microscope (Olympus Corporation, Tokyo, Japan), and photographed. For invasion determination, the Matrigel membrane (BD Biosciences) was inserted for pre-coating.

### Wound healing assay

Cell migration capacity was examined using the scratch assay. In brief, 3×10^5^ cells were inoculated into the six-well plate to incubate overnight until confluence. A scratch was then gently made in the middle of cells using a pipette tip. Cells were washed twice with PBS, and serum-free medium was added to the culture for an additional 24 h. Subsequently, images were captured, and cell migration was determined. The coverage rate of cells with a scratch wound was calculated using the Image-Pro Plus software (version 6.0; Media Cybernetics, CA, USA).

### Colony formation assay

Monoclonal formation was detected through colony analysis. Cells were inoculated into the six-well plate (10^3^ cells/well) for 6 h. Fresh medium was then added into each well for 10 days to support cell growth and colony formation. The surviving colonies were stained with 0.1% crystal violet, and the colony number in each well was calculated. Three independent experiments were carried out.

### Xenograft experiment

The animal experiments were approved by the Ethics Committee of The First Hospital of Lanzhou University, and all animal procedures were carried out in strict accordance with the Guidelines for Animal Use in the National Institutes of Health. Six-week-old male BALB/C-Null nude mice (Cancer Institute of the Chinese Academy of Medical Science, Shanghai, China) were used for the xenograft experiment. A549 cells in which LDH-A was stably silenced down and the blank control group were generated by transfection with small hairpin RNAs (shRNAs; Genepharma, China). Cells from the sh-LDH-A group and sh-NC group (1×10^6^ cells/well) were inoculated subcutaneously into nude mice. After injection, the subcutaneous tumor volume in nude mice was measured and calculated every 4 days according to the following formula: *V* = 0.5 × length × width^2^. Four weeks later, the nude mice were killthe present studyed, and the tumors were removed for measurement and photographing.

### Statistical methods

The relationships between LDH-A expression and the clinicopathological features of patients were analyzed using the chi-square test. Pairwise comparison was carried out by Student’s *t*-test. Additionally, the Kaplan–Meier method was employed for plotting the patient survival curve, and the survival rate between two groups was compared using the Log-rank test. The primary outcome indexes included disease-free survival (DFS) and overall survival (OS). Moreover, the risk factors affecting the prognosis for LUAD patients were predicted using the Cox proportional hazard model. The SPSS 19.0 software (IBM, Armonk, NY, USA) was utilized for statistical analysis. A difference of *P* < 0.05 was deemed statistically significant.

## Results

### LDH-A expression in LUAD tissues

**Figure 1:**
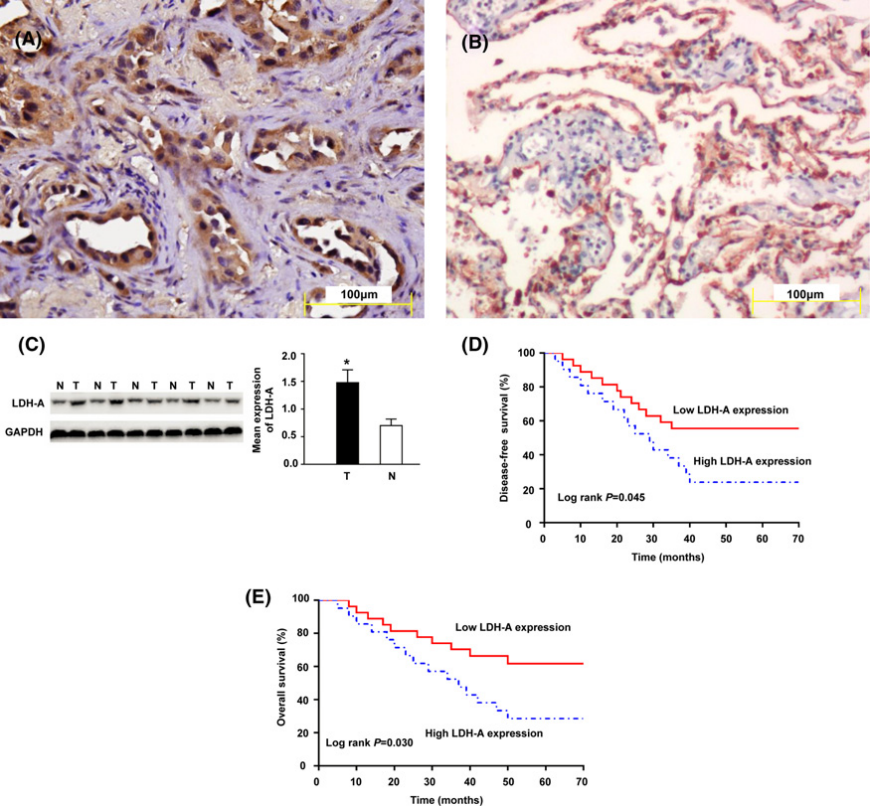
The LDH-A protein expression in LUAD tissues The LDH-A protein expression in cytoplasm of LUAD (**A**) and in cytoplasm of para-carcinoma tissues (**B**) which was detected by IHC; scale bars set at 100 µm. The expression levels of LDH-A proteins in LUAD tissues and normal lung tissues by using the Western blotting (**C**). Kaplan–Meier DFS (**D**) and OS (**E**) curve of LUAD patients correlated with LDH-A expression; high LDH-A expression means worse prognosis in patients with LUAD. **Note:** LDH-A means lactate dehydrogenase A; LUAD means lung adenocarcinoma; IHC means immunohistochemistry; N means normal lung tissues; T means tumor tissues; DFS means disease-free survival; OS means overall survival. * *P* < 0.05.

According to the IHC results, LDH-A protein was mainly located in the cytoplasm of LUAD cells (Figure 1A,B). In the 50 LUAD tissues, the high LDH-A expression rate was 46.0% (23/50), while the high LDH-A expression rate in para-carcinoma tissues was 24.0% (12/50). The difference between the two groups was statistically significant (*P* = 0.021). To verify the above results, we quantitatively detected LDH-A expression levels in 50 LUAD and para-carcinoma tissue pairs by Western blotting. The results indicated that LDH-A expression level in cancer tissues was remarkably higher than para-carcinoma tissues, and the difference was statistically significant (*P* < 0.05; Figure 1C).

### Relationship between LDH-A and LUAD prognosis

Fifty LUAD patients were divided into low LDH-A expression (*n* = 27) and high expression (*n* = 23) groups according to the IHC results. In addition, the relationships between LDH-A expression and the clinicopathological features of LUAD patients were analyzed. It was discovered that high LDH-A expression was closely related to the lymph node status and TNM stage (*P* < 0.05); however, it was not related to the age, sex, smoking history, tumor size, and differentiation degree of LUAD patients (*P* > 0.05). The above data are summarized in Table 1. To explore the relationships between LDH-A expression status and the prognosis of LUAD patients, all patients were followed up for an extended period, and the Kaplan–Meier survival curve was plotted. The results suggested that the DFS and OS in the high LDH-A expression group were shorter than those in the low expression group (Figure 1D,E). Moreover, all variables affecting the OS of LUAD patients were analyzed using the Cox proportional hazard model, and the single-factor analysis results suggested that prognosis of LUAD patients closely correlated with the LDH-A expression status (Table 2). Specifically, a positive lymph node, advanced TNM stage, and high LDH-A expression were indicative of poor prognosis for LUAD patients. Multiple-factor analysis demonstrated that TNM stage and LDH-A expression were independent factors in predicting the prognosis of LUAD patients (Table 2).

**Table 1:** Correlations between LDH-A expression and clinicopathological features in LUAD patients

Clinicopathologic characteristics	*n*	LDH-A expression (%)		X^2^	*P* value
		Low	High		
Total	50	27 (54.0%)	23 (46.0%)		
Sex				2.566	0.109
Male	31	14 (45.2%)	17 (54.8%)		
Female	19	13 (68.4%)	6 (31.6%)		
Age (years)				0.809	0.368
≥60	27	13 (48.1%)	14 (51.9%)		
<60	23	14 (60.9%)	9 (39.1%)		
Smoking				1.469	0.226
Yes	28	13 (46.4%)	15 (53.6%)		
No	22	14 (63.6%)	8 (36.4%)		
Differentiation				1.422	0.491
Well	8	3 (37.5%)	5 (62.5%)		
Middle	15	8 (53.3%)	7 (53.8%)		
Poor	27	16 (59.3%)	11 (40.7%)		
Tumor size (cm)				1.239	0.266
≥3	24	11 (45.8%)	13 (54.2%)		
<3	26	16 (61.5%)	10 (38.5%)		
Lymph node metastasis				8.566	0.003
Yes	28	10 (35.7%)	18 (64.3%)		
No	22	17 (77.3%)	5 (22.7%)		
TNM stage				4.903	0.027
I+II	34	22 (64.7%)	12 (35.3%)		
III	16	5 (31.3%)	11 (68.7%)		

**Note:** LDH-A means lactate dehydrogenase A; LUAD means lung adenocarcinoma; TNM means tumor node metastasis.

**Table 2:** Univariate and multivariate analysis of the association of prognosis with clinicopathologic parameters and LDH-A expression in LUAD patients

Characteristic	Univariate analysis			Multivariate analysis		
	HR	*P* value	95%CI	HR	*P* value	95%CI
Sex (male vs female)	1.112	0.915	0.593–1.629			
Age (≥60 vs <60 years)	0.993	0.745	0.532–1.397			
Smoking (yes vs no)	0.589	1.092	0.728–1.993			
Differentiation (well vs middle vs poor)	1.396	0.081	1.283–2.117			
Tumor size (≥3 cm vs <3 cm)	1.121	0.369	0.889–1.796			
Lymph node metastasis (yes vs no)	1.574	0.032	0.991–2.076	1.297	0.093	1.022–1.952
TNM stage (I+II vs III)	1.655	0.027	1.215–1.978	1.733	0.039	1.231–1.993
LDH-A expression (low vs high)	1.407	0.043	0.885–1.850	1.488	0.047	1.002–1.937

**Note:** LDH-A means lactate dehydrogenase A; LUAD means lung adenocarcinoma; HR means hazard ratio; CI means confidence interval; TNM means tumor node metastasis.

### siRNA-mediated knockdown of LUAD cell line

**Figure 2:**
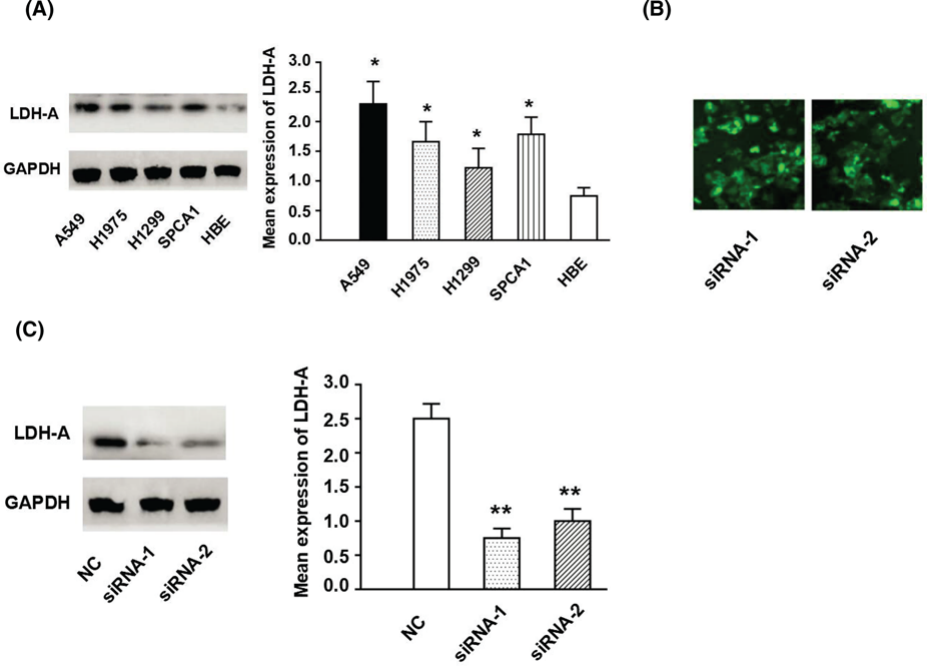
The LDH-A protein expression in LUAD cell lines (**A**) The expression levels of LDH-A proteins in LUAD cell lines (A549, H1975, H1299, and SPCA1) and HBE by using the Western blotting. (**B**) Transfection efficiency was observed by fluorescence microscopy. (**C**) The cells were transfected with the siRNA, the expression levels of LDH-A protein in LUAD cells line A459 was assessed by Western blotting. **Note:** LDH-A means lactate dehydrogenase A; LUAD means lung adenocarcinoma; HBE means human bronchial epithelial cell line; NC means normal control, untreated A549 cell lines. * *P* < 0.05; ** *P* < 0.01.

The LDH-A expression levels in LUAD cell lines (including A549, H1975, H1299, and SPCA1), as well as in the normal bronchial epithelial cell line HBE, were analyzed by Western blotting. Amongst these, the LDH-A expression level was the highest in the A549 cell line (Figure 2A). To explore the role of LDH-A in LUAD, LDH-A expression in A549 cells was knocked down using siRNA. After transfecting the cells with siRNA, green fluorescence was observed in the cytoplasm, which indicated satisfactory transfection results (Figure 2B). The results of Western blotting indicated that, compared with the A549 cells in the NC group, LDH-A expression of the siRNA-1 and siRNA-2 groups was markedly suppressed (Figure 2C). Therefore, they could be used for subsequent experiments.

### Effect of siRNA-mediated LDH-A knockdown in the LUAD cell line

**Figure 3:**
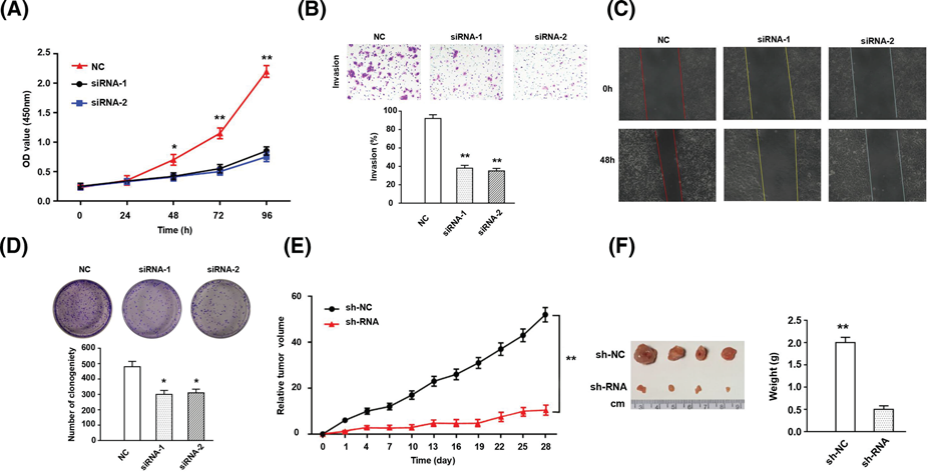
The effection of LDH-A on biological behavior of A549 cell lines (**A**) Cell proliferation was determined with an CCK-8 assay; cells were transfected with LDH-A siRNA and absorbance was detected at 24 h, 48 h, 72 h, and 96 h, post transfection. (**B**) Cell invasion ability was assessed by transwell; the lower column graph indicates the quantity of invasive cells. (**C**) Cell migration capacity was examined using the wound healing assay; the percentage of migration closure rate was calculated. (**D**) Monoclonal formation was detected through colony analysis; the lower column graph indicates number of clones after 10 days. (**E**) The subcutaneous tumor volume in nude mice was measured and calculated every 4 days. (**F**) Four weeks later, the nude mice were killed, and the tumors were taken out for measurement. **Note:** NC means normal control, untreated A549 cell lines; LDH-A means lactate dehydrogenase A. * *P* < 0.05; ** *P* < 0.01.

After effectively knocking down LDH-A expression in the LUAD cell line A549, the effect of LDH-A on A549 cells was detected through different experimental approaches. Through CCK-8 kit detection, it was discovered that suppressed LDH-A expression dramatically reduced the proliferation capacity of A549 cells (Figure 3A). In the transwell assay, LDH-A knockdown restrained the invasion capacity of A549 cells (*P*< 0.01; Figure 3B). In addition, the scratch assay demonstrated that siRNA-mediated LDH-A silencing lowered the migration capacity of the LUAD A549 cells (Figure 3C). Furthermore, colony formation ability was one of the standards used to evaluate tumor cell proliferation activity. The colony formation experiment proved that the colony formation ability of A549 cells in the NC group was higher than that in the siRNA-LDH-A group (*P*< 0.05; Figure 3D). Similar to the CCK-8 results, effective suppression of LDH-A expression markedly reduced the proliferation activity of A549 cells.

### LDH-A suppressed tumorigenicity of LUAD cells *in vivo*

To further verify the potential role of LDH-A in LUAD growth, we constructed a xenograft model *in vivo*. A549 cells were injected subcutaneously into nude mice to further detect the tumor weight and volume. After successful injection of A549 cells, the subcutaneous tumor volume in nude mice was measured and calculated every 4 days. The results indicated that knocking down LDH-A expression suppressed the subcutaneous growth of A549 cells in nude mice, but it did not completely alter tumorigenesis (*P* < 0.01; Figure 3E). Compared with the sh-NC group, tumors resected from nude mice in sh-LDH-A groups were markedly smaller (*P* < 0.01; Figure 3F). In conclusion, LDH-A could promote tumorigenicity in LUAD cells.

### Effect of LDH-A on the epithelial–mesenchymal transition (EMT) of LUAD cells

**Figure 4:**
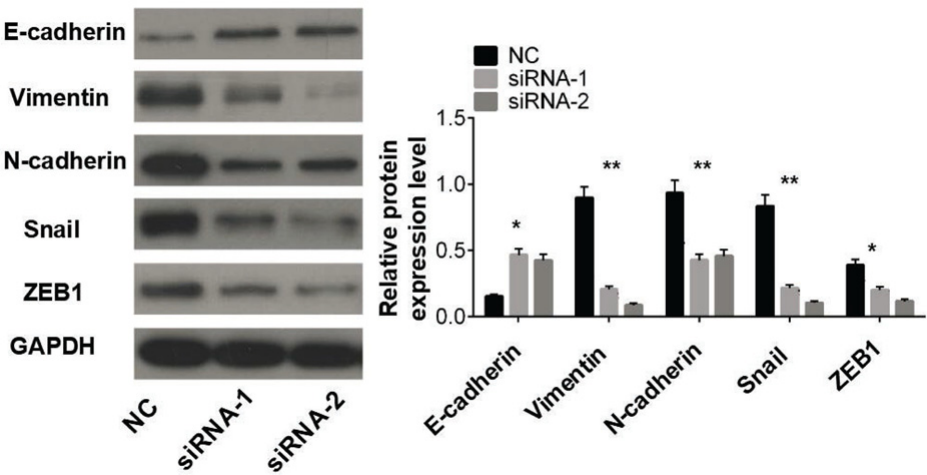
Down-regulating LDH-A expression would suppress the EMT process in LUAD cells **Note:** LDH-A means lactate dehydrogenase A; EMT means epithelial-mesenchymal transition; LUAD means lung adenocarcinoma. * *P* < 0.05; ** *P* < 0.01.

Epithelial–mesenchymal transition (EMT) was the major malignant phenotype of tumor cells, which endowed tumor cells with invasive and metastatic capacities. Consequently, changes in the expression of EMT-related proteins in A549 cells before and after LDH-A knockdown were detected. The results suggested that the expression level of E-cadherin protein was markedly up-regulated, while those of Vimentin, N-Cadherin, Snail, and ZEB1 were distinctly down-regulated (*P* < 0.05, Figure 4). Thus, it could be concluded that down-regulating LDH-A expression may suppress the EMT process in LUAD cells.

## Discussion

Diagnostic and treatment technologies for LUAD patients have continuously developed. In particular, the development and extensive clinical application of novel targeted medicines have effectively improved the survival time of LUAD patients [8]. However, the prognosis for advanced LUAD patients remains dismal, and its 5-year survival rate is lower than 10% [9]. Therefore, searching for a new therapeutic target is a current research focal point. Previous studies reveal that cancer cells show high glucose uptake capacity, accompanied by mitochondrial respiratory injury [10]. Consequently, the phenomenon of generating self-required energy through anaerobic glycolysis is referred to as the Warburg effect [10–12]. Thus, searching for an antitumor therapeutic target based on the abnormal energy metabolism of cancer cells has become a new research focus.

LDH-A is one of the important isozymes of lactate dehydrogenase, which is involved in cell energy metabolism. Numerous studies have indicated that LDH-A is abnormally expressed in multiple solid tumor cells, revealing that LDH-A plays a crucial role in maintaining the biological behaviors of cancer cells [7,13–16]. In lymphoma, suppressing LDH-A can reduce the ATP level in cancer cells and induce their apoptosis [17]. Furthermore, several studies have verified that LDH-A also plays a key role in kidney cancer, liver cancer, breast cancer, nasopharyngeal carcinoma, and pancreatic cancer [7,18–20]. LDH-A is a key enzyme in anaerobic glycolysis and can be converted into lactic acid, as catalyzed by pyruvic acid [21]. When LDH-A is suppressed, more pyruvic acid will enter the tricarboxylic acid (TCA) cycle, which requires more oxygen. However, cancer cells are excessively dependent on aerobic glycolysis, in which ATP can be produced quickly and more precursors are available to satisfy the metabolic requirements for rapid proliferation [21]. Thus, the TCA cycle and subsequent mitochondrial oxidative phosphorylation (OXPHOS) pathway are frequently impaired and dysfunctional.

In the present study, the LDH-A expression level in LUAD tissues was first detected using clinical samples, and the results suggested that LDH-A was up-regulated in LUAD tissues. Moreover, LDH-A was markedly correlated with the lymph node status and TNM stage of LUAD patients, revealing that LDH-A plays a critical role during the genesis and development of LUAD. Second, the LUAD patients were followed up for an extended period, and it was discovered that LUAD patients with high LDH-A expression had shorter survival times and poorer prognosis. To verify the above clinical phenomena, we constructed the LDH-A down-regulation LUAD cell model using siRNA to further detect the malignant phenotypes, such as proliferation, invasion, and migration. The results suggested that suppressing LDH-A could reduce the proliferation, invasion, and migration capacities of LUAD cells. Similar conclusions could be drawn from the xenograft experiment in nude mice.

Nonetheless, the mechanism by which LDH-A participates in LUAD progression remains unclear. EMT is proposed to be a key step during tumor cell metastasis. During the process of EMT initiating tumor cell invasion and metastasis, cell polarity is lost. This reduces contact with the surrounding matrix, while the cell migration and mobility capacities are markedly enhanced, finally forming the metastatic lesion [22,23]. EMT causes loss of polarity in epithelial cells, which then separate or detach from the epithelial tissue and move to other positions. In the present study, we have preliminarily verified that LDH-A can regulate EMT in LUAD cells, which may be involved in cancer cell invasion and migration.

LDH-A can promote the malignant biological behaviors of LUAD cells, and is a promising target for LUAD treatment. However, the pathway and mechanism by which LDH-A promotes LUAD genesis and development remain unclear, and should be further investigated in future studies.

## Abbreviations

AJCC: American Joint Committee on Cancer
DFS: disease-free survival
EMT: epithelial–mesenchymal transition
FBS: fetal bovine serum
HBE: human bronchial epithelial cell line
IHC: Immunohistochemistry
LUAD: lung adenocarcinoma
LDH-A: lactate dehydrogenase A
NC: normal control group
OS: overall survival
PBS: phosphate buffer solution
PVDF: polyvinylidene fluoride
UICC: Union for International Cancer Control
SDS-PAGE: sodium dodecyl sulfate- polyacrylamide gel electrophoresis
shRNAs: small hairpin RNAs
siRNA: small interference RNA
TNM: tumor node metastasis
