# Correction: LDH-A promotes malignant behavior via activation of epithelial-to-mesenchymal transition in lung adenocarcinoma

**DOI:** 10.1042/BSR-20181476_COR

**Published:** 2020-06-26

**Keywords:** LDH-A, LUAD, Prognosis, Tumor progression, EMT

The authors of the original article above article “LDH-A promotes malignant behavior via activation of epithelial-to-mesenchymal transition in lung adenocarcinoma” (Biosci Rep (2019) 39(1), https://doi.org/10.1042/BSR20181476) would like to correct Figure 3C, as the image of 0h for siRNA-1 and siRNA-2 due to mis-operation in visualisation. The authors declare that these corrections do not change the results or conclusions of their paper, and express their sincere apologies for any inconvenience that this error has caused to the readers. The corrected version of Figure 3 is presented here.

**Figure 3:**
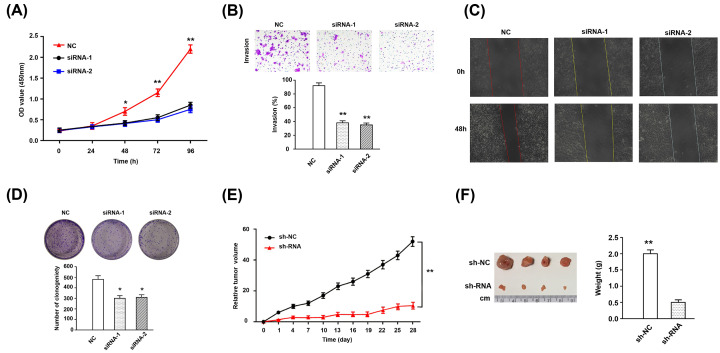
The effection of LDH-A on biological behavior of A549 cell lines (**A**) Cell proliferation was determined with an CCK-8 assay; cells were transfected with LDH-A siRNA and absorbance was detected at 24 h, 48 h, 72 h, and 96 h, post transfection. (**B**) Cell invasion ability was assessed by transwell; the lower column graph indicates the quantity of invasive cells. (**C**) Cell migration capacity was examined using the wound healing assay; the percentage of migration closure rate was calculated. (**D**) Monoclonal formation was detected through colony analysis; the lower column graph indicates number of clones after 10 days. (**E**) The subcutaneous tumor volume in nude mice was measured and calculated every 4 days. (**F**) Four weeks later, the nude mice were killed, and the tumors were taken out for measurement. **Note:** NC means normal control, untreated A549 cell lines; LDH-A means lactate dehydrogenase A. *P < 0.05; **P < 0.01.

